# The Risk of Cardiovascular Diseases in Axial Spondyloarthritis. Current Insights

**DOI:** 10.3389/fmed.2021.782150

**Published:** 2021-11-08

**Authors:** Eric Toussirot

**Affiliations:** ^1^INSERM CIC-1431 Centre Investigation Clinique, CHU de Besançon, Besançon, France; ^2^Rhumatologie, CHU de Besançon, Besançon, France; ^3^Département de Thérapeutique, Université de Bourgogne Franche-Comté, Besançon, France; ^4^INSERM UMR1098 Relations Hôte Greffon Tumeurs, ingénierie cellulaire et génique, Université de Bourgogne Franche-Comté, Besançon, France

**Keywords:** ankylosing spondylitis, spondyloarthritis, cardiovascular risk, cardiovascular risk factors, atherosclerosis

## Abstract

There is an increased cardiovascular (CV) risk in axial spondyloarthritis (axSpA), leading to increased CV mortality and morbidity in these patients. The factors that may explain this enhanced CV risk in axSpA are multiple, including traditional CV risk factors such as smoking, but also the inflammatory process and probably the use of non-steroidal anti-inflammatory drugs (NSAIDs). The CV involvement of axSpA may be detected at an early and pre-clinical stage, using non-invasive techniques. While NSAIDs play a deleterious role in the CV risk of axSpA, TNF inhibitors seem to have a beneficial impact, but this remains to be demonstrated in specific clinical studies. More data are needed to determine the potential effects of IL-17 inhibitors on the CV risk of axSpA. CV comorbidity has been mainly assessed in the radiographic form of axSpA, while limited data are available in patients with the non-radiographic form. The current management of axSpA must consider this CV comorbidity according to the EULAR recommendations. Rheumatologists play a determinant role in the detection of CV risk and current management of these patients is focused on the control of disease activity, suppression of inflammation, screening for and management of traditional CV risk factors, as well as the restriction of NSAID use.

## Introduction

Axial spondyloarthritis (axSpA) refers to an interrelated group of inflammatory rheumatic diseases (IRD) that primarily affect the axial skeleton (1). Radiographic changes of the sacroiliac joints are a hallmark of the disease, and enable the diagnosis of ankylosing spondylitis (AS) according to the modified New York criteria (2). The Assessment of SpondyloArthritis international Society (ASAS) has developed a set of criteria for the recognition of patients with early axSpA that includes evidence of sacroiliitis visible by magnetic resonance imaging (MRI), chronic back pain, HLA-B27 positivity and other non-articular symptoms (3). These criteria distinguish the radiographic (r-axSpA, formely AS) and non-radiographic (nr-axSpA) forms of axSpA, according to the presence or absence of structural changes of the sacroiliac joints on pelvic X-rays. When the clinical features predominate in peripheral joints, SpA may be classified as a peripheral form (pSpA).

Besides extra-articular manifestations (psoriasis, acute anterior uveitis and inflammatory bowel diseases), additional comorbidities are described in axSpA, including cardiovascular (CV) involvement (4, 5). Rheumatoid arthritis (RA) is another IRD for which there is ample evidence of an increased CV risk (6). In parallel, there is a cumulating body of evidence that axSpA may promote the development of atherosclerosis, CV manifestations and complications including myocardial infarction and/or stroke (4, 5, 7).

In this review, we analyze the evidence for CV mortality and morbidity in axSpA, as well as the traditional CV risk factors that may participate in the increased CV risk seen in axSpA patients.

## Mortality in axSpA

Mortality in axSpA is mostly described in AS, with limited information in nr-axSpA, due to the recent individualization of this subgroup (4, 8). This excess mortality in AS has been previously related to radiation treatment, a therapeutic option that was used until the seventies (9). However, studies on the mortality rate in AS are not concordant. In an analysis from the Mayo Clinic (Rochester, Minnesota USA) in 1979, there was no difference in mortality between male patients with AS and the general male population (10). On the contrary, other studies have reported an excess of mortality in AS, with a standardized mortality ratio (SMR) ranging from 1.6 to 1.9 (11–13) (Table 1). These studies took radiation therapy into account, and the increased mortality persisted even in patients who did not receive this treatment. A relationship between mortality in AS and disease duration and severity was also reported (13). A study from Norway confirmed these results (14): in a population of 677 AS patients followed in a reference center for a 34 year period, the SMR was increased in male patients [1.63; 95% confidence interval (CI): 1.29–1.97] compared to a control group. Circulatory disease was the most frequent cause of death (40%) and the factors linked to decreased survival were delayed diagnosis, an increase in acute phase reactants, work disability and the absence of NSAID use. In a series of 2,154 patients with AS from Hong Kong, the SMR was calculated to be 3.07 [2.64–3.5], and 1.89 [1.61–2.13] after adjustment for age. The most frequent cause of death was infection, ahead of CV complications (15). In nationwide cohorts of AS patients from Scandinavian countries, the (age- and sex-) adjusted hazard ratio (HR) for death among AS patients was 1.60 [1.44–1.77] with increased mortality in both male and female patients. A low level of education and general comorbidities (including CV diseases) were identified as predictors of death (16). In a Spanish study analyzing CV mortality and CV events at 5 years in different IRD, AS was found to have the highest risk of a first CV event [HR: 4.6 (1.32–15.99)], but without increased CV deaths (17). Finally, a recent meta-analysis concluded that CV mortality in AS was increased, with a relative risk (RR) of 1.46 [1.15–1.86] (18).

**Table 1:** Standardized mortality ratio (SMR) or hazard ratio in patients with radiographic axial spondyloarthritis.

**References**	**Number of patients**	**Country**	**Duration of follow-up (years)**	**SMR/HR**
Smith and Doll (9)	14,111	United Kingdom	16	SMR: 1.66
Radford et al. (11)	836	United Kingdom	13	SMR: 1.6 (men)
Karprove et al. (12)	151	Canada	27	SMR1.93 (men)
Lethinen et al. (13)	398	Finland	25.7	SMR:1.5
Bakland et al. (14)	677	Norway	33	SMR: 1.63 (men)
Mok et al. (15)	2,154	China	9	SMR: 1.88
Exarchou et al. (16)	8,600	Sweden	6	HR: 1.6

## Cardiovascular Morbidity in axSpA

Mortality in axSpA (AS) is consistently related to CV diseases and complications. In a large American administrative database comparing CV diseases in different IRD including 1,843 patients with AS, the prevalence ratios for ischemic heart disease, cerebrovascular disease and congestive heart failure ranged between 1.2 and 1.8 (19). In a retrospective cohort study conducted in Canada, and including 8,616 patients with AS, the prevalence of CV and cerebrovascular diseases increased with age, but age- and sex-stratified prevalence ratios were highest in younger people, ranging from 1.25 for cerebrovascular disease to 1.37 for ischemic heart disease (20). These results were in line with recent data from a Swedish cohort followed from 2006 to 2012, where the standardized incidence ratios (SIRs) for acute coronary syndrome and stroke were higher in patients compared to the general population (4.3 and 5.4/1,000 person years compared to 3.2 and 4.7, respectively) (21). On the contrary, the study by Brophy et al. (22) did not find an increased prevalence of acute myocardial infarction or stroke among patients with AS compared to those without AS. Different meta-analyses have examined the risk of CV diseases and/or complications in patients with axSpA. Mathieu *et al* performed such an analysis in 2011, and updated it in 2015. In the first meta-analysis, based on 11 studies, there was no significant increase in myocardial infarction or stroke in AS (23), while in the updated meta-analysis, which included 6 additional studies, there was a significant increase in both CV complications [myocardial infarction: odds ratio (OR) 1.6 (1.32–1.93) and stroke: OR 1.5 (1.39–1.62), respectively] (24). The more recent meta-analysis of 16 studies by Kim *et al* also reported a significantly higher risk of myocardial infarction in AS [RR: 1.49 (1.34–1.66) (18). The COMOSPA study was devoted to comorbidity analysis in SpA, including patients with axial or peripheral SpA (ax-SpA: 89%; pSpA: 56%]. The prevalence of ischemic heart disease and cerebrovascular disease was 2.7% [2.2–3.2] and 1.3% [0.9–1.7], respectively (25). Finally, results form a cross-sectional study in Spain were recently published. That study examined the CV burden in patients with r-axSpA and nr-axSpA by means of ultrasound assessment. The results indicated that the atherosclerotic involvement was similar between these 2 forms (26).

## Cardiovascular Risk Factors in axSpA

There are several possible explanations for the higher CV risk in axSpA: inflammation is a well-recognized factor for accelerated atherosclerosis in different IRD, including axSpA, while traditional CV risk factors may also play a role. In addition, specific axSpA-related cardiac manifestations (aortic insufficiency and atrioventricular conduction disturbances) may also contribute to the enhanced CV risk.

### Smoking

It is now established that cigarette smoking has a negative influence on axSpA activity and severity (27). In the French DESIR cohort of patients with early SpA, smoking was associated with early disease onset, high disease activity, increased axial structural damage on X-ray, and poor quality of life (28). In the COMOSPA study, the prevalence of smoking in SpA ranged from 6 to 44%, depending on the countries (25).

### Hypertension

It is estimated that the prevalence of hypertension is higher in axSpA than would be expected in the general population (4). In a small series of patients with AS (*N* = 20), 20% had hypertension (29). In the COMOSPA study, the proportion was higher (33.5%), especially in northern European countries (25). Continuous use of NSAIDs may impact on the development of hypertension: in a prospective longitudinal r-axSpA cohort, continuous NSAID use was associated with a 12% increased risk for the development of incident hypertension compared to patients with non-continuous use or no NSAID use (30).

### Diabetes and Metabolic Syndrome

In the COMOSPA study, the prevalence of diabetes was 8.8% (25). A high frequency of metabolic syndrome according to the NCEP-ATP III criteria was found in a limited series of patients with axSpA (*N* = 24; frequency: 45.8 vs. 10.9% in the controls) (31).

### Obesity

A recent study found that patients with axSpA may present with increased weight: in that study, obesity was found in 22% of patients and overweight in 37%, according to the WHO criteria (32). However, body composition studies did not find an excess of fat mass, but an increased lean mass (33).

### Dyslipidemia

The relationships between lipid parameters and IRD are complex. Indeed, inflammation has a wide range of effects on lipids, both quantitatively and qualitatively. Under the pressure of inflammation, both LDL and HDL cholesterol tend to decrease, but with an imbalanced atherogenic index (total cholesterol/HDL cholesterol) (34). In parallel, lipids are subject to qualitative changes, toward a pro-atherogenic profile for HDL cholesterol, and oxidation for the LDL fraction (35). All these modifications promote an increased CV risk in IRD (36). In axSpA, similar lipid changes are described. The prevalence ratio of dyslipidemia was 1.2 in a large series of patients with AS (19). In more recent series, HDL cholesterol was found to be decreased, with an elevated atherogenic index (37, 38). In addition, the levels of HDL cholesterol and apolipoprotein-A have been associated with CV complications in axSpA [HR: 3.67 (1.47–9.06) and 1.89 (1.02–3.54)] (39).

## Preclinical Atherosclerosis in axSpA

Non-invasive techniques may be used for the identification of early arterial wall changes in IRD. Ultrasonography of carotid arteries is the most widely used method, giving results on intima media thickness (IMT), pulse wave velocity (PWV) and flow mediated dilatation (FMD). It is a valid method for the identification of pre-clinical atherosclerosis with the capacity to predict future CV disease and complications (7). This method has been extensively used in RA, showing impaired endothelial function as well as arterial wall changes (40). Similar results were obtained in patients with axSpA. In two meta-analyses, IMT was reported to be increased in patients with AS (23, 41). A third recent meta-analysis of 35 studies reported an increased IMT and PWV and a decreased FMD in patients with AS, indicative of a higher risk of subclinical atherosclerosis (42). Carotid plaques are frequently observed in axSpA compared to a control population (29.7 vs. 9.4%) and the presence of such plaques correlated with acute phase reactants but not IMT (43). Endothelial dysfunction as evaluated by FMD was impaired in a series of 43 patients with axSpA compared to 40 healthy controls (44). In the cross-sectional study from Spain, there was no difference in the prevalence of carotid plaques or in carotid IMT between patients with r-axSpA and those with nr-axSpA. In addition, when using the CV risk assessment model (SCORE), the percentage of patients classified in the very high risk category was comparable between the 2 groups, indicating that the CV burden was similar in nr-axSpA and r-axSpA (26).

## The Effect of Treatment on Cardiovascular Risk in axSpA

The CV impact of the treatments that are routinely used in axSpA may be bidirectional, i.e., beneficial and/or detrimental. Indeed, since atherosclerosis should be seen as an inflammatory disease, and the CV risk in IRD is largely influenced by inflammation, controlling inflammation would have a favorable impact on this comorbidity. In RA, it is well-established that methotrexate (MTX) and TNFα inhibitors (TNFi) positively influence the CV risk (45).

### NSAIDs

It is well-recognized that all the NSAIDs have a negative impact on CV risk, contributing to an excess of CV complications (46). Among the different NSAID class, coxib and diclofenac were associated with the higher risk of major vascular events while this risk was less with naproxen (46). NSAIDs are used as first line of treatment in axSpA, but not continuously, according to different recommendations. However, the CV risk induced by NSAIDs in axSpA remains controversial. Indeed, in a Canadian study on the mortality of AS, the lack of NSAID intake was one factor associated with increased CV mortality (47). The same result was found in a Norwegian study showing an excess of death from vascular disease. One of the factors associated with reduced life expectancy was the lack of NSAID use [OR: 4.53; (1.75–10.77)] (14). To explain these results, it was suggested that NSAIDs may partially control inflammation, and thus, in patients not using NSAIDs, atherosclerosis may accelerate. However, these results may reflect a selection bias by proposing NSAIDs only to patients without CV risk factors.

### Conventional Synthetic DMARDs

Sulfasalazine (SLZ) is used in selective cases of SpA, with limited efficacy in the peripheral form. Since SLZ has an aspirin moiety, it has been suggested that this drug may have a protective CV effect. In a Taiwanese population-based retrospective study, the use of SLZ provided a protective effect against CV diseases in patients with AS [HR: 0.65 (0.43–0.99)] (48).

### Biological DMARDs

In RA, TNFi result in a 20–50% reduction in CV risk (49). There are numerous studies demonstrating that TNFi may reduce subclinical inflammation in axSpA. Results are not universally concordant, but overall, TNFi may favorably impact IMT and endothelial dysfunction, with a stabilizing effect on the atheromatous plaque (50–52). In the systematic review by Tam et al. it was concluded that TNFi are effective in AS in preventing or even reversing the progression of IMT in patients responding to treatment. Pulse wave velocity was reduced or unchanged under TNFi treatment, while aortic augmentation index was also largely unchanged (53). Lipid parameters may fluctuate under TNFi. In axSpA, the atherogenic index did not change under infliximab, while it improved under etanercept (54, 55). In a prospective study of patients with AS receiving a TNFi, we did not observe significant changes in insulin parameters (HOMA-IR index) (56).

The experience with IL-17 inhibitors (IL-17i) in axSpA is shorter compared to the use of TNFi, with no specific study examining the CV risk. It is considered that IL-17A may have deleterious effect on the arterial wall and cardiac cells, with potentially pro-atherogenic properties (57). In the secukinumab and ixekizumab development programs, there were no reports of increased CV events (58, 59). Similarly, the long term follow-up of patients with psoriasis under secukinumab did not reveal a specific CV signal or an increase in major adverse CV events (MACE) or CV mortality (60, 61). Major CV events were not increased with long term use of ixekizumab in a pooled analysis from 21 clinical trials in patients with psoriasis, psoriatic arthritis (PsA) and axSpA (62).

### Targeted Synthetic DMARDs

Janus kinase inhibitors (JAKi) (tofacitinib, upadacitinib, filgotinib) are currently in development in axSpA, but upadacitinib has recently been approved for the treatment of axSpA. During the phase 3 clinical trials, there was no safety signal regarding CV events with JAKi in patients with axSpA (63). In a systematic review and meta-analysis of the CV effects of JAKi in patients with RA in randomized controlled trials, there was no evidence of a significant change in CV risk in the short term (64). In a *post hoc* analysis from the RA, psoriasis and PsA development programs and observational studies, the incidence rates for deep vein thrombosis (DVT), pulmonary embolism (PE) and venous thromboembolism (VTE) were similar between tofacitinib doses (5 vs. 10 mg twice daily) but generally higher in patients with CV or VTE risk factors (65). However, concerns have recently emerged regarding the risk of thrombosis under tofacitinib, leading to warnings by competent authorities. The ORAL surveillance trial analyzed the safety of tofacitinib (5 mg and 10 mg twice daily) vs. a TNFi in subjects with RA aged 50 years or older and with at least one additional CV risk factor. The primary endpoints in this trial were non-inferiority of tofacitinib compared to TNFi in regard to MACE and malignancies (excluding non-melanoma skin cancer). Final analysis of ORAL surveillance showed that the non-inferiority criteria were not met for the primary comparison of the combined tofacitinib doses vs. TNFi [HR for MACE: 1.33 (0.91–1.94); HR for malignancies: 1.48 (1.04–2.09)] ^1^. The conclusion is that there was a higher risk of MACE and malignancies (excluding non-melanoma skin cancer) with tofacitinib as compared to TNFi in patients with RA. Following these results, healthcare professionals were advised to keep considering the benefits and risks of tofacitinib when deciding to prescribe and continue patients on the drug ^2^.

## Discussion

**Figure 1:**
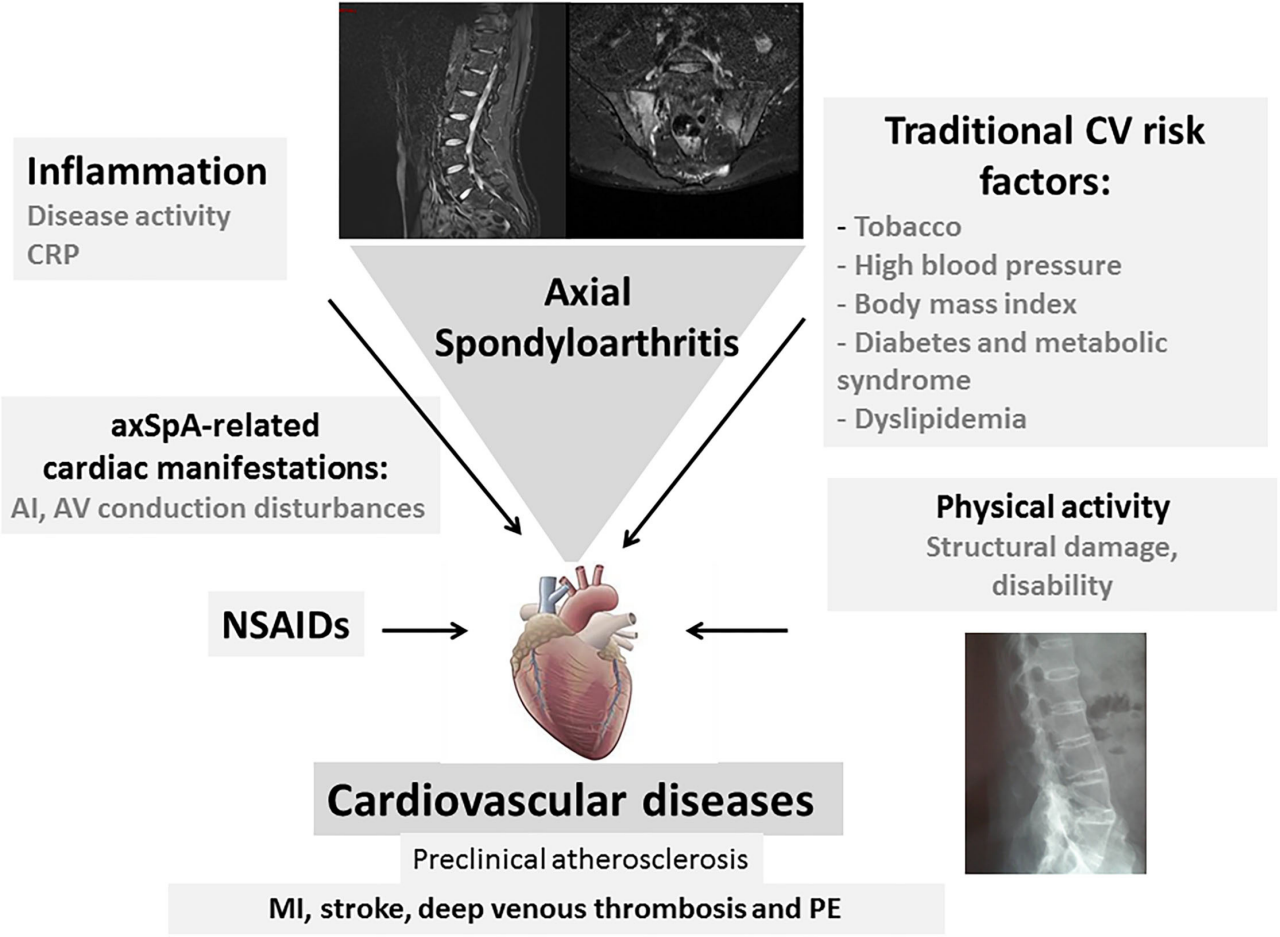
Cardiovascular (CV) complications in axial spondyloarthritis (axSpA). Cardiovascular events that are observed in axSpA are related to traditional CV risk factors and the inflammatory process. They are linked to CV mortality and morbidity. Specific cardiac manifestations such as aortic insufficiency and atrioventricular conduction disturbances contribute to this CV involvement in patients with axSpA. Long-term NSAID use may accentuate this CV risk in axSpA. The structural damage and loss of mobility contribute to the CV risk (CV, cardiovascular; CRP, C-reactive protein; AI, aortic insufficiency; NSAIDs, non-steroidal anti-inflammatory drugs; PE, pulmonary embolism).

There is compelling evidence that there is an increased CV risk in axSpA, and that it represents a major comorbidity deserving specific attention. In a Swedish study comparing the CV events between AS, RA and the general population, the adjusted RR for stroke was equivalent between AS and RA [1.5 (1.1–2.0) and 1.5 (1.2–1.8), respectively] while there was a smaller increase for acute coronary syndrome in RA than in AS [1.7 (1.4–2.0) and 1.3 (1.0–1.7), respectively]. For thromboembolic events, the risk was also less in AS compared to RA (66). Similar to RA, the increased CV risk in axSpA may be detected at a preclinical stage using non-invasive techniques (43) (Figure 1). It is well known that inflammation is a major determinant of atheroma development, from plaque initiation to thrombosis. It has been established from epidemiological studies that high sensitivity CRP levels predict CV events in the general population, leading to a higher CV risk to that induced by LDL cholesterol during atheroma formation (67). Inflammation in axSpA may be noticed by elevated circulating high sensitivity CRP, interleukin-6 or homocysteine (68). The EULAR recommendations for the management of patients with axSpA include the “abrogation of inflammation,” highlighting the need to control inflammation to reach this goal (69). The parameters evaluating arterial wall dysfunction in axSpA correlated with disease activity or severity measurements. In addition, it should be stressed that CV risk has mainly been evaluated in the radiographic form of axSpA, whereas limited data exist in nr-axSpA. Recommendations for the CV risk management in RA have been elaborated by the EULAR group and expanded to axSpA and PsA (70). General principles for the detection and management of CV diseases in axSpA are outlined, including the correct use of NSAIDs. The identification and subsequent management of traditional CV risk factors is also important. The guidelines recommend the use of risk prediction algorithms, such as SCORE to determine which patients require lipid-lowering and anti-hypertensive therapies. For RA, it is recommended to adjust available prediction scores such as SCORE, by a multiplication factor of 1.5 in order to appropriately evaluate the 10 year CV risk. However, in axSpA, this adjustment is not validated. Lifestyle recommendations include the benefits of a healthy diet, regular exercise and smoking cessation. Physical activity may benefit patients with axSpA, reducing not only CV risk factors, but also disease activity (71).

## Conclusion

Similarly to RA, CV risk is increased in axSpA. This comorbidity is a reality for patients with axSpA and must be adequately evaluated. It is highly probable that the use of bDMARDs (TNFi and probably IL-17i) and specific treatment strategies, such as the treat-to-target paradigm should have a favorable impact on CV risk, and thus, disease prognosis. However, specific clinical studies examining this question are required. Overall, CV risk detection and management is an important aspect for patients with axSpA that deserves specific attention from physicians.

## Author Contributions

The author confirms being the sole contributor of this work and has approved it for publication.

## Conflict of Interest

The author declares that the research was conducted in the absence of any commercial or financial relationships that could be construed as a potential conflict of interest.

## Publisher's Note

All claims expressed in this article are solely those of the authors and do not necessarily represent those of their affiliated organizations, or those of the publisher, the editors and the reviewers. Any product that may be evaluated in this article, or claim that may be made by its manufacturer, is not guaranteed or endorsed by the publisher.

## Footnotes

^1^https://clinicaltrials.gov: NCT02092467.

^2^https://www.pfizer.com/news/press-release/press-release-detail/pfizer-shares-co-primary-endpoint-results-post-marketing.
